# Resilience of anaerobic digestion to polypropylene microplastic contamination: Kinetic and structural evidence

**DOI:** 10.1371/journal.pone.0346428

**Published:** 2026-04-03

**Authors:** Napapat Sitthikitpanya, Alissara Reungsang

**Affiliations:** 1 Department of Biotechnology, Faculty of Technology, Khon Kaen University, Khon Kaen, Thailand; 2 Research Group for Development of Microbial Hydrogen Production Process from Biomass, Khon Kaen University, Khon Kaen, Thailand; 3 Academy of Science, Royal Society of Thailand, Bangkok, Thailand; Luleå University of Technology, SWEDEN

## Abstract

The increasing occurrence of microplastics (MPs) in organic waste streams raises concerns about their impact on anaerobic digestion (AD). This study examined the effect of polypropylene MPs (PP-MPs) on methane production during AD of food waste for 150 days under batch conditions. PP-MPs were added at 10–300 mg g^-1^ total solids (TS), covering reported MP levels in food waste and food packaging materials and extending to worst-case scenarios. Methane yields ranged from 310.2 to 324.4 mL CH_4_ g^-1^ volatile solids (VS) across treatments versus 334.3 ± 5.2 mL CH_4_ g^-1^ VS in the control, with no significant differences (*p* = 0.634). Kinetic modeling confirmed no consistent inhibitory trends. FTIR and SEM analyses indicated minor surface oxidation and cracking on PP-MPs, while the polymer backbone remained intact, suggesting only superficial aging. These results provide critical assurance for waste-to-energy facilities processing plastic-contaminated organic waste streams. Although PP-MPs do not impair AD performance, their persistence and potential fragmentation pose environmental risks. These findings provide critical insight into the resilience of AD systems and emphasize the need for strategies to mitigate secondary MP formation in biogas production from contaminated waste streams.

## Introduction

Microplastics (MPs), defined as plastic particles smaller than 5 mm, are generated through the chemical, physical, and biological degradation of plastic products [1]. These particles can persist in the environment for centuries and tend to accumulate across diverse ecosystems. In 2019, global plastic production reached approximately 460 million tons, generating over 353 million tons of plastic waste [2]. Alarmingly, more than 60% of this waste was mismanaged, leading to environmental leakage, widespread plastic pollution, and ecological risks for various organisms [3,4].

The focus on polypropylene MPs (PP-MPs) is particularly critical as PP is one of the most widely used polymers in food packaging and household products due to its high mechanical strength, resistance to puncture, and low moisture permeability [5]. This leads to frequent detection of PP-MPs in municipal solid waste and organic waste streams including food waste (FW) [1,3], raising urgent questions about their interactions with biological treatment processes in industrial anaerobic digestion (AD) facilities.

Previous studies have shown that polystyrene (PS)-MPs can significantly inhibit methane production during AD of FW [6]. Li et al. [7] reported that PS-MPs delay hydrolysis, cause volatile fatty acid accumulation, induce the generation of reactive oxygen species (ROS), and suppress key enzymatic activities, ultimately reducing methane yield. Similarly, Zhang et al. [8] found that MPs such as polyvinyl chloride (PVC), polypropylene (PP), and polyhydroxyalkanoate (PHA) reduced cumulative methane production by 9.69%, 5.52%, and 14.48%, respectively, whereas polyethylene (PE) increased methane yield by 11.97% compared to the control. Moreover, Yang et al. [9] demonstrated that polyethylene terephthalate (PET)-MPs exerted size-dependent effects on methane production during sludge digestion, with 300–500 µm particles inhibiting methane production by 11.3–24.9%. These findings indicate that the impacts of MPs on AD performance are highly variable and context-dependent, rather than universally inhibitory. The effects may differ depending on experimental and operational conditions. Key influencing factors include polymer type, particle size, concentration, and surface properties. These characteristics can alter microbial activity, enzyme interactions, and substrate accessibility during AD. Several studies have reported inhibitory effects of PS-, PVC-, and PET-MPs on microbial activity and methane production. However, a critical knowledge gap exists regarding PP-MPs, despite their prevalence in food packaging. Conflicting reports in the literature show that Zhang et al. [8] reported a 5.52% reduction, while Xiao et al. [10] reported a 10.8% enhancement in methane production by PP-MPs. These discrepancies may result from differences in MP dosage, particle size distribution, substrate composition, and microbial community structure. Therefore, a systematic evaluation under controlled conditions is required.

Therefore, this study aims to provide definitive assessment of PP-MPs impact on methane production during AD of FW at concentrations ranging from 10–300 mg g^-1^ total solids (TS) over 150 days, simulating both typical and extreme contamination scenarios. The comprehensive approach combining kinetic modeling with structural characterization (FTIR and SEM) provides critical guidance for industrial AD operations managing increasingly plastic-contaminated organic waste streams.

## Materials and methods

### Substrate and inoculum preparation

The synthetic FW was prepared to represent the typical composition of household organic waste in Thailand, based on a modified formulation from Moonsamy et al. [11]. The mixture, on a wet weight basis, consisted of 26.2% vegetables, 26.2% fruits, 14.5% grains, 14.2% meat, 8.85% rice, 8.85% noodles, and 1.2% cooking oil. The moisture content was adjusted to 85% using distilled water. The components were then finely ground and thoroughly mixed to ensure homogeneity. The homogenized mixture was subsequently heated in a water bath at 90 °C for 1 h as a thermal hygienization pretreatment. Finally, the slurry was filtered through a 2-mm mesh sieve to remove large particles, improve substrate homogeneity, and ensure stable operation of the laboratory-scale AD system. The prepared FW contained 19.71 ± 0.18% TS, 19.50 ± 0.17% volatile solids (VS), 80.29 ± 0.18% moisture, and 1.07 ± 0.03% ash (all w/w), with a carbon-to-nitrogen (C/N) ratio of 20.66. The substrate was stored at −20 °C until use.

Anaerobic granules were used as the inoculum for methane production. They were obtained from a digester at the internal circulation (IC) wastewater treatment facility of Khon Kaen Brewery Co., Ltd., Khon Kaen, Thailand. Prior to use, the IC granules were washed twice with tap water and degassed under anaerobic conditions for 3 days. The inoculum was then stored at 4 °C until use. The inoculum contained 7.00 ± 0.04% TS, 5.89 ± 0.04% VS, 93.00 ± 0.04% moisture, and 15.79 ± 0.35% ash (all w/w).

### Microplastic preparation

PP-MPs were prepared according to the procedure described by Choonut et al. [12]. Briefly, PP was purchased from Sigma-Aldrich (St. Louis, Missouri, USA), ground using a freezer mill, and sieved to obtain particles smaller than 5.0 mm. The resulting PP-MPs were translucent white and irregularly shaped. Sterilization of the PP-MPs was performed by immersion in 70% ethanol for 45 min, followed by drying at 50 °C for 24 h and exposure to ultraviolet (UV) light at a wavelength of 254 nm for 15 min.

#### Biochemical methane potential (BMP) test of different concentrations of PP-MPs.

The BMP tests were conducted in 120 mL glass serum bottles with a working volume of 70 mL. The synthetic FW and IC granules were added at an inoculum-to-substrate ratio (ISR) of 2:1 based on VS [13,14]. The PP-MPs were then introduced at concentrations of 10, 50, 100, 200, and 300 mg g^-1^ TS of the substrate. This concentration range was selected to encompass reported levels of MPs in FW and food packaging materials [1,7,15], while higher concentrations were included as worst-case scenarios to assess the influence of PP-MP concentration on AD performance. A control treatment containing FW and inoculum without PP-MPs was prepared. An inoculum blank (inoculum only) was included to account for background methane production. The initial pH was adjusted to 7.0 ± 0.2 using 5 M NaOH or HCl. All bottles were purged with nitrogen gas for 5 min to ensure anaerobic conditions and then sealed with rubber stoppers and aluminum crimp caps. The tests were incubated at 35 °C and agitated at 150 rpm for 150 days. The incubation period was selected to ensure that methane production had stabilized and that no further significant increase in cumulative methane yield was observed [16]. In addition, the incubation time was extended to assess potential long-term effects of PP-MPs on AD performance. All experimental setups were performed in quadruplicate. During the fermentation, the volume and composition of biogas were monitored using a wetted glass syringe method [17] and gas chromatography (GC) [18], respectively. Biogas volume was measured daily, and the produced gas was released after each measurement to avoid pressure accumulation. After 150 days of fermentation, the digestate was collected and the residual PP-MPs were manually separated. The recovered PP-MPs were washed with distilled water and rinsed with 70% ethanol to remove adhering organic matter and residual microbial biomass, then dried in a hot air oven at 50 °C for 24 h [12]. The dried samples were subsequently analyzed by Fourier-transform infrared spectroscopy (FTIR) and scanning electron microscopy (SEM) to assess potential physicochemical changes.

### Analytical methods and kinetic modeling

The TS and VS contents were determined according to standard analytical procedures [19]. The initial pH was measured using a digital pH meter (pH-500, Queen, USA). Methane content was analyzed using GC (Shimadzu GC-2014, Tokyo, Japan), and its cumulative volume was calculated based on the mass balance method described by Zheng and Yu [20]. Methane yield, expressed as mL CH_4_ g^-1^ VS, was calculated by subtracting the cumulative methane production of the inoculum blank from that of the substrate prior to normalization by the VS of the substrate added [16]. Kinetic parameters were estimated by fitting cumulative methane production data to the modified Gompertz model [21], expressed as Eq. (1):


$$P(t)=\;P_{max}exp\left\{-exp\left[\frac{R_{m}e}{P_{max}}(\lambda \;-\;t)\;+\;\text{1}\right]\right\}$$ (1)

where *P*(t) is the cumulative methane production at time *t* (mL CH_4_ g^-1^ VS), *P*_max_ is the maximum methane potential (mL CH_4_ g^-1^ VS), *R*_m_ is the maximum methane production rate (mL CH_4_ g^-1^ VS d^-1^), λ is the lag phase (d), and *e* is Euler’s number (2.7183).

Model performance was evaluated by residual analysis, where residuals were calculated as the difference between observed and model-predicted methane yields. The distribution of residuals over digestion time was examined to assess the adequacy of model fitting (S1 Fig).

The structural alterations of PP-MPs were assessed through functional group analysis using FTIR spectroscopy (Tensor II, Bruker, Germany) equipped with a Platinum ATR unit, covering the spectral range of 4000–400 cm^-1^ [22]. For SEM analysis, PP-MP samples before and after fermentation were washed with distilled water to remove impurities, oven-dried at 50 °C for 24 h, and then mounted on aluminum stubs using conductive double-sided carbon tape. To prevent charging effects during imaging, the samples were demagnetized and sputter-coated with a thin gold layer prior to examination by a LEO 1450VP SEM operated at 10.0 kV to observe surface morphological changes resulting from degradation [12].

### Statistical analysis

Statistical analyses were performed using IBM SPSS Statistics, Version 28 (IBM Corp., Armonk, NY, USA). Data are presented as mean ± standard deviation (SD). The normality of data distribution in each group was assessed using the Shapiro–Wilk test, and homogeneity of variances was evaluated using Levene’s test. Differences among experimental groups were analyzed using one-way analysis of variance (ANOVA). When significant differences were detected, Duncan’s multiple range test was conducted for post hoc comparisons among group means. A *p*-value < 0.05 was considered statistically significant.

## Results and discussion

### Effects of PP-MPs on methane production and kinetics

**Fig 1:**
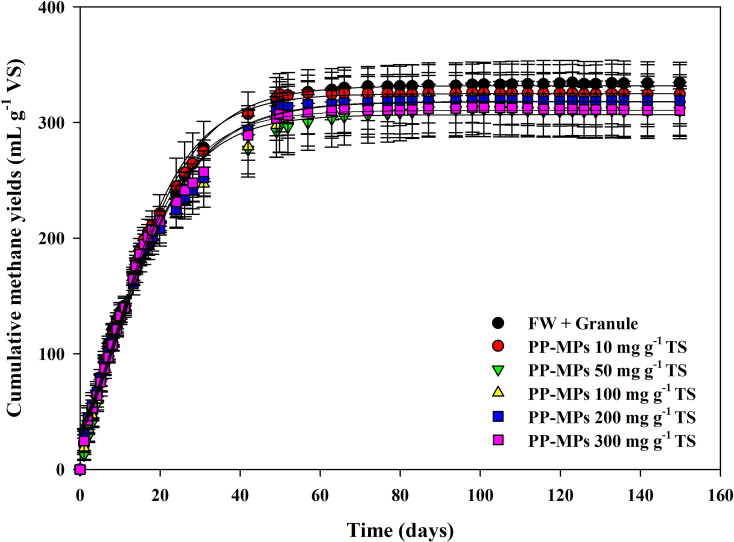
Cumulative methane yield profiles during batch anaerobic digestion of food waste (FW) with varying concentrations of polypropylene microplastics (PP-MPs) (10–300 mg g^-1^ TS).

Fig 1 illustrates the cumulative methane production profiles during AD of FW with PP-MPs at concentrations from 10–300 mg g^-1^ TS. The methane yield of the control treatment (without PP-MPs) was 334.3 ± 5.2 mL CH_4_ g^-1^ VS, whereas treatments containing PP-MPs showed slightly reduced yields ranging from 310.2 to 324.4 mL CH_4_ g^-1^ VS. However, one-way ANOVA indicated no statistically significant effect of PP-MPs on methane production under the experimental conditions tested (*p* = 0.634). These results indicate that PP-MP contamination did not significantly affect methane production under the experimental conditions, even at concentrations higher than those typically reported in FW and mechanically depackaged FW (approximately 0.001–0.6% w/w) [1,15], during a prolonged digestion period of 150 days.

The kinetic analysis derived from the modified Gompertz model (Table 1) corroborated these observations. The potential methane production (P) for the control was 331.8 ± 1.6 mL CH_4_ g^-1^ VS, while treatments with PP-MPs ranged from 306.8 to 324.9 mL CH_4_ g^-1^ VS. The greatest reduction was observed at 50 mg g^-1^ TS (306.8 ± 1.9 mL CH_4_ g^-1^ VS); however, the overall differences among treatments were below 7.5% and not statistically significant (one-way ANOVA, *p* = 0.712). Similarly, the maximum methane production rate (R_m_) and lag phase (λ) exhibited no consistent inhibitory trend with increasing PP-MP concentration. All treatments exhibited similar maximum Rₘ of 9.8–11.4 mL CH_4_ g^-1^ VS d^-1^) and consistently negative lag phase values (λ = −2.6 to −1.4 d), indicating immediate methane generation without any detectable startup delay. These negative λ values are characteristic of BMP assays involving highly biodegradable substrates, where methanogenic activity proceeds faster than the Gompertz model typically predicts [23]. FW is particularly well-suited for this accelerated biogas production due to its favorable composition and physical characteristics. The substrate’s high moisture content and rich concentrations of readily hydrolysable carbohydrates, proteins, and lipids require minimal enzymatic pretreatment, enabling direct utilization by anaerobic microorganisms [24,25]. It should be noted that the FW used in this study was filtered through a 2-mm mesh sieve prior to digestion, which differs from typical full-scale AD practice. This particle-size reduction may have further enhanced substrate accessibility and hydrolysis, thereby contributing to the high methane yields and negative lag-phase values observed. This immediate microbial activity reflects two important conditions: first, the exceptional biodegradability of the FW substrate, and second, the effective acclimation of the anaerobic microbial community to the experimental environment. Critically, the consistent performance across all treatments demonstrates that PP-MPs did not negatively impact AD under these conditions.

**Table 1:** Kinetic parameters estimated using the modified Gompertz model for batch anaerobic digestion of food waste (FW) with different concentrations of polypropylene microplastics (PP-MPs).

Treatment	P (mL CH_4_ g^-1^ VS)	R_m_ (mL CH_4_ g^-1^ VS d^-1^)	λ (days)	R^2^
FW + Granule (Control)	331.8 ± 1.6	10.7 ± 0.3	−1.8 ± 0.4	0.9947
PP-MPs 10 mg g^-1^ TS	324.9 ± 1.3	11.4 ± 0.3	−1.4 ± 0.3	0.9960
PP-MPs 50 mg g^-1^ TS	306.8 ± 1.9	10.6 ± 0.4	−1.5 ± 0.5	0.9909
PP-MPs 100 mg g^-1^ TS	317.6 ± 2.2	9.8 ± 0.4	−2.4 ± 0.6	0.9892
PP-MPs 200 mg g^-1^ TS	318.2 ± 1.7	9.9 ± 0.3	−2.6 ± 0.5	0.9933
PP-MPs 300 mg g^-1^ TS	310.5 ± 1.4	10.8 ± 0.3	−1.6 ± 0.4	0.9948

P: Methane yield, R_m_: Maximum methane production rate, λ: Lag-phase time, R^2^: Coefficient of determination.

The negligible effect of PP-MPs on AD can be attributed to the inherent physicochemical properties of PP, which include high hydrophobicity, water insolubility, and low density [26]. These characteristics caused PP particles to aggregate and float at the surface rather than disperse uniformly within the fermentation medium, as observed during the experiment, thereby limiting their interaction with anaerobic microorganisms compared to other types of MPs. Furthermore, PP is considered an inert polymer with an extremely low tendency to bind biomolecules [27], which likely prevents disruption of microbial metabolic pathways essential for AD. In contrast, other polymers such as PVC have been reported to release toxic compounds like bisphenol A (BPA) [28], while PET can leach harmful chemicals such as di-n-butyl phthalate, both of which significantly inhibit hydrolysis and methanogenesis [29]. Additionally, exposure to MPs such as PS, PE, and PET has been reported to induce the generation of ROS in microbial cells, leading to oxidative stress and subsequent inhibition of methanogenic activity [3,30]. Interestingly, Xiao et al. [10] observed that the addition of PP-MPs at a concentration of 300 particles g^-1^ total suspension solids increased methane production by 10.8% compared to control digesters, which was attributed to enhanced microbial abundance rather than inhibition. These findings demonstrate that PP-MPs exert no adverse effects on AD performance or stability and, under certain conditions, may even promote methane production, highlighting their minimal inhibitory potential compared to other MP types.

The practical implications of these findings are significant for waste-to-energy facilities and AD operations. Unlike previous studies demonstrating substantial methane production inhibition from PS-MPs (11.3–24.9% reduction reported by Yang et al. [9]) and notable reductions from PVC (9.69%) and PHAs (14.48%) as reported by Zhang et al. [8], PP-MPs did not significantly affect AD performance under the experimental conditions, even at high contamination levels. This finding is particularly relevant as AD technology serves as a cornerstone of the circular bioeconomy, where plastic contamination of organic waste streams is increasingly common. The demonstrated resilience of AD systems to PP-MP contamination indicates that methane production performance may remain stable under PP-MP contamination. However, management strategies for plastic separation should consider the downstream handling and application of digestate, in order to minimize potential environmental risks related to MP persistence and fragmentation.

### FTIR and SEM characterization of microplastic particles

**Fig 2:**
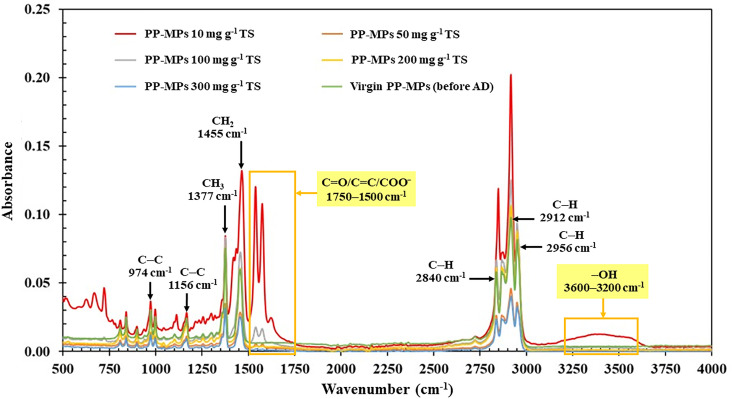
Overlaid Fourier-transform infrared (FTIR) spectra of virgin polypropylene microplastics (PP-MPs, before anaerobic digestion) and PP-MPs after anaerobic digestion at different concentrations (10–300 mg g^-1^ TS).

FTIR and SEM analyses provided mechanistic insights into the interaction between PP-MPs and the AD environment. The FTIR spectrum of virgin PP-MPs (Fig 2, green line) exhibited characteristic absorption bands of PP, including C − H stretching vibrations at 2956, 2912, and 2840 cm^-1^, CH_2_ bending at 1455 cm^-1^, CH_3_ bending at 1377 cm^-1^, and C–C stretching vibrations at 1156 and 974 cm^-1^ [31]. After digestion, spectral alterations were observed in the 1750–1500 cm^-1^ and 3600–3200 cm^-1^ regions, corresponding to C = O/C = C/COO⁻ stretching and broad –OH stretching vibrations, respectively [32]. These changes suggest the formation of oxygen-containing functional groups via surface oxidation under AD conditions, consistent with previous reports on weathered and biologically degraded PP [32,33]. Importantly, the persistence of the original hydrocarbon-related bands indicates that the polymer backbone remained largely intact, with degradation primarily confined to surface-level modification rather than extensive chain scission or bulk depolymerization [33].

Notably, the intensity of the oxidation-related bands did not increase proportionally with increasing PP-MP dosage. In ATR-FTIR analysis, spectral intensity is influenced by contact between particles and the ATR crystal, as well as by particle surface morphology [34,35], in addition to concentration effects. Therefore, the relatively stronger oxidation signals observed at lower dosages likely reflect more efficient surface exposure and surface-level modification rather than a direct concentration-dependent response.

**Fig 3:**
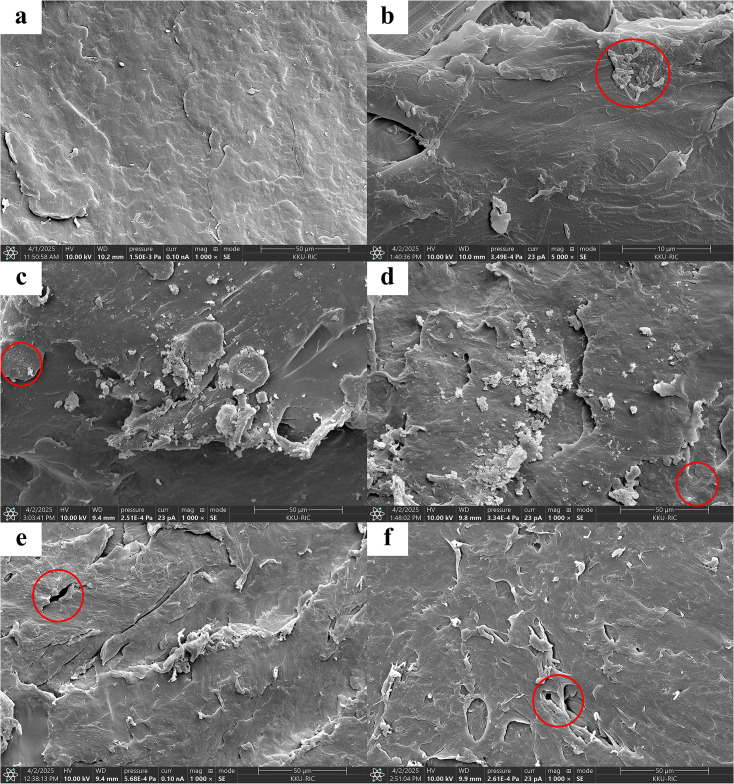
Scanning electron microscopy (SEM) images of polypropylene microplastics (PP-MPs) before and after 150 days of anaerobic digestion of food waste: (a) pristine PP-MPs with a smooth surface (1000 × , 50 µm scale bar); (b) 10 mg g^-1^ TS showing biofilm formation and microbial attachment (indicated by red circles) (5000 × , 10 µm scale bar); (c) 50 mg g^-1^ TS and (d) 100 mg g^-1^ TS showing small surface pits (indicated by red circles) (1000 × , 50 µm scale bar); (e) 200 mg g^-1^ TS and (f) 300 mg g^-1^ TS showing surface pits and cracks (indicated by red circles) (1000 × , 50 µm scale bar).

SEM images supported the FTIR findings by revealing surface alterations of PP-MPs during AD (Fig 3). Pristine particles exhibited a smooth surface (Fig 3a), whereas digested PP-MPs showed biofilm formation and microbial attachment (Fig 3b), along with the development of small pits and surface cracks (Figs 3c–f). These morphological changes correspond to oxidative reactions indicated by FTIR but remain restricted to the particle surface. The combination of chemical stability and limited surface erosion explains the negligible effect of PP-MPs on methane production during AD, as their inert nature limits interaction with the microbial consortia.

Although PP-MPs do not impair AD performance, their persistence and breakdown into smaller particles may still pose environmental risks. Potrykus et al. [36] reported that PP recovered from a municipal landfill after approximately five years showed surface weathering and MP formation, while the bulk polymer remained largely intact. These findings highlight the long-term stability of PP under landfill conditions and suggest that it undergoes gradual fragmentation rather than complete degradation. From an environmental perspective, partial surface aging could enhance fragmentation, generating secondary MPs during sludge handling or land application of digestate. These smaller fragments may exhibit greater mobility and sorption capacity for co-contaminants such as heavy metals, antibiotics, or persistent organic pollutants, thereby increasing ecological risks. Consequently, integrated management strategies—including upstream plastic reduction, digestate post-treatment, and MP monitoring—are essential to mitigate downstream impacts while supporting circular economy frameworks.

## Conclusions

This study demonstrated that PP-MPs exert no significant impact on methane production during AD of FW, even at high PP-MP concentrations and extended incubation time (150 days). Methane yield and kinetics exhibited no inhibitory trends. FTIR and SEM analyses revealed only minor surface oxidation and cracking without polymer backbone alteration. These findings confirm AD system resilience and minimal inhibitory potential. While PP-MPs do not compromise AD performance, their persistence and fragmentation risk underscore the need for strategies to mitigate secondary MP formation within circular bioeconomy systems. Future studies should explore synergistic effects with co-contaminants, microbial dynamics, and long-term accumulation to ensure sustainability of AD in plastic-polluted environments.

## Supporting information

S1 Fig Residual plot of the modified Gompertz model for cumulative methane production. Residuals were calculated as the difference between observed and model-predicted methane yields.
(TIF)
